# Daily mood and cognitive performance of women with and without bipolar disorder: role of menopausal status

**DOI:** 10.1007/s00737-023-01359-4

**Published:** 2023-09-04

**Authors:** Yanlin Liu, Hui Xin Ng, Federica Klaus, Jared W. Young, Lisa T. Eyler

**Affiliations:** 1 Department of Psychology, University of California San Diego, San Diego, CA USA; 2 Department of Cognitive Science, University of California San Diego, San Diego, CA USA; 3 Department of Psychiatry, University of California San Diego, San Diego, CA USA; 4 Desert-Pacific Mental Illness Research Education and Clinical Center, VA San Diego Healthcare System, San Diego, CA USA

**Keywords:** Bipolar disorder, Menopausal status, Daily mood, Attentional performance

## Abstract

We examined the role of menopausal status in daily mood and cognitive performance among women with bipolar disorder (BD) compared to healthy comparison women. We analyzed the association of menopausal status, bipolar diagnosis, and their interaction on daily mood assessed by mobile surveys and attentional performance measured multiple times over 2 weeks. Menopausal status was associated with more daily negative affect in women with BD, but not related to attentional performance.

## Introduction

Sex differences have been observed in bipolar disorder (BD), a psychiatric disease characterized by alternating depression and mania and cognitive deficits. Compared to men, women with BD experience more depressive symptoms, more rapid cycling, and a higher rate of suicide (Perich et al. 2021), suggesting a possible role for reproductive hormones. Given the major hormone changes during menopause, it is surprising that few studies have examined how pre- and post-menopausal BD women differ in mood and cognition. BD patients experience more severe and chronic depression and higher anxiety peri-and post-menopause than those cycling (pre-menopausal) (Perich et al. 2021). Attention is one of the most affected cognitive domains in BD; complaints of concentration are commonly found in women during menopausal transition, associated with objective cognitive decline as well (Hogervorst et al. 2022).

Most studies used retrospective designs, with data collected at one timepoint, making it unclear if changes in mood/cognition occur. Few examined mood ratings or attention over time in women with BD in different reproductive stages. Ecological momentary assessment (EMA), a powerful technique to collect data about real-time mood states in daily settings, though used in women with premenstrual dysphoric disorder (Beddig et al. 2020), has not been used to understand the relationship of female reproductive status to mood in BD. Furthermore, despite evidence that the number and severity of prior mood episodes were strong indicators of cognitive impairments in BD (Burdick et al. 2022), the association between daily mood and cognition has not been assessed in the context of menopausal status in BD.

We examined mood, attention, and reproductive status in women with and without BD aged 25–60 years cross-sectionally. Women with BD were expected to have less positive and more negative daily affect and worse attention compared to HC women. In BD and HC, though lesser in HC, we hypothesized post-menopausal women would have less positive and more negative daily affect and worse attention than pre-menopausal women. We expected less positive and more negative mood to relate to poorer attention, particularly in BD.

## Methods

Data came from first-year visits of a longitudinal study of cognitive aging in BD and HC. Pregnant women, individuals younger than 18 years, and non-English speakers were excluded. In an initial visit, all subjects completed diagnostic and clinical interviews and self-rated their sleep quality (PSQI, Pittsburgh Sleep Quality Index; Buysse et al. 1989; higher scores = worse sleep). Trained raters determined that all patients met DSM-IV diagnostic criteria for BD or were free of any diagnosis (HC) and rated depressive (Hamilton Depression Rating Scale; Hamilton 1960) and manic (Young Mania Rating Scale; Young et al. 1978) symptom severity.

Participants received surveys three times daily—morning, early afternoon, and evening—delivered to their own mobile device via SMS (text) messaging over 14 days. At in-person visits at the beginning, middle, and the end of this period, they completed a 5-choice computerized continuous performance test (5C-CPT; Bhakta and Young 2017). Data from 86 participants who were women and completed at least 14 mobile surveys (minimum one survey per day), minimum one assessment of attention, and provided covariate data were analyzed. Participants were self-declared as cycling or post-menopausal using the Menstrual History Questionnaire, adapted from Roy-Byrne et al. (1986).

Mobile self-report surveys evaluated momentary affect for 7 items on a scale of 1, “not at all,” to 7 “very much.” Ratings were combined into positive (“energetic,” “confident,” and “happy”) and negative (“sad or depressed,” “angry or upset,” “anxious or nervous,” and “stressed”) affect scores. We calculated means across all surveys during the 2-week period. Participants completed 14 to 42 (mean $\pm$ SD: 33 $\pm$ 6) surveys.

5C-CPT requires participants to respond to visual target stimuli with a button press while withholding response for non-targets. A sensitivity index (SI) was calculated based on hit rate (proportion correct responses to targets) and false alarm rate (proportion responses to non-targets) and averaged across the three administrations.

Analyses were performed using Statistical Package for Social Sciences 28. Continuous demographic variables were compared using general linear model, while the categorical race/ethnicity was compared using binary regression. General linear models examined group, menopausal status, and a group x menopausal interaction as predictors of the mean SI or mean affect scores. Age, years of education, race/ethnicity, and PSQI scores were included as covariates to control for group differences in our sample and based on literature linking sleep changes to menopause and to mood (Lee et al. 2008; Perich et al. 2021). For mood composites with a significant group x menopausal status interaction, follow-up models examined individual affect ratings. We reran mood models after removing outliers to see if results were held. We also examined the relationship of positive and negative affect to SI and whether this differed by diagnostic group, reproductive status, or diagnostic group x reproductive status interaction. Level of significance was alpha = 0.05 (two-sided).

## Results

Of the 86 women included, 47 were cycling (19 BD) and 39 were menopausal (17 BD). Of the cycling group, 7 BD and 4 HC participants reported irregular cycling, and 2 BD and 1 HC were over 50 years old, suggesting possible peri-menopausal status. As expected, compared to cycling, menopausal women had a higher average age (*p* < 0.001, Table 1). HC completed more years of education than BD patients (*p* = 0.002). BD women had higher average PSQI scores (*p* < 0.001), indicating worse sleep, and lower global cognitive T-score (*p* < 0.001) compared to HC. BD women showed higher variability in both mood and attention than HC groups.

**Table 1:** Participant characteristics

	HC-cycling		HC-menopause		BD-cycling		BD-menopause		General linear model^a^												Summary
									Diagnostic group				Reproductive group				Interaction				
	*N*	Mean ± SD / %	*N*	Mean ± SD / %	*N*	Mean ± SD / %	*N*	Mean ± SD / %	F	df	*p*	η^^2^	F	df	*p*	η^^2^	F	df	*p*	η^^2^	
Demographics																					
Age at visit	28	43.0 ± 6.4	22	54.9 ± 4.7	19	40.7 ± 7.9	17	56.0 ± 4.0	0.215	1	0.644	0.003	106.861	1	< 0.001	0.566	1.597	1	0.210	0.019	BD = HC, C < M, no significant int^g^
Education (mean years)	27	16.1 ± 1.7	22	15.4 ± 1.7	18	14.6 ± 1.8	16	14.4 ± 2.2	9.809	1	0.002	0.110	1.305	1	0.257	0.016	0.487	1	0.487	0.006	BD < HC, C = M, no significant int
Race/ethnicity																					BD = HC, C = M, no significant int^h^
African American	6	21.4%	3	13.6%	2	10.5%	3	17.6%													
Asian	1	3.6%	2	9.1%	2	10.5%	–	–													
White	11	39.3%	12	54.5%	12	63.2%	9	52.9%													
Hispanic	10	35.7%	4	18.2%	3	15.8%	3	17.6%													
Native Pacific Islander	–	–	1	4.5%	–	–	1	5.9%													
Other	–	–	–	–	–	–	1	5.9%													
Sleep quality (PSQI)^b^	26	4.5 ± 1.7	18	4.8 ± 2.9	19	8.7 ± 4.7	16	9.8 ± 3.0	41.667	1	< 0.001	0.357	1.019	1	0.316	0.013	0.241	1	0.625	0.003	BD > HC, C = M, No Significant Int
Global cognitive *T*-score^c^	28	53.2 ± 9.0	20	51.2 ± 9.1	18	43.4 ± 9.3	16	44.0 ± 7.3	17.796	1	< 0.001	0.186	0.003	1	0.954	0.000	0.507	1	0.479	0.006	BD < HC, C = M, No Significant Int
Clinical variables																					
Age of onset	–	–	–	–	10	17.9 ± 5.0	15	19.6 ± 12.0													
Depression severity (HAM-D)^d^	–	–	–	–	18	14.1 ± 10.2	17	20.8 ± 9.7													
Mania severity (YMRS)^e^	–	–	–	–	18	5.1 ± 4.7	17	6.1 ± 5.2													
Number of medications																					
Psychiatric	25	0.0 ± 0.0	21	0.1 ± 0.5	19	2.4 ± 1.8	17	2.6 ± 1.2													
Antipsychotic	28	0.0 ± 0.0	22	0.1 ± 0.4	19	0.6 ± 0.6	17	0.5 ± 0.5													
Non-psychiatric	9	2.3 ± 2.1	10	2.7 ± 1.2	12	1.9 ± 1.0	12	3.3 ± 2.5													
Currently on hormone medication	0	–	0	–	1	–	1	–													
Mean affect																					
Positive affect	28	5.41 ± 0.89	22	5.63 ± 0.78	19	4.54 ± 0.67	17	4.30 ± 1.04	34.669	1	< 0.001	0.297	0.003	1	0.955	0.000	1.507	1	0.223	0.018	BD < HC, C = M, no significant int
Energetic	28	4.73 ± 1.02	22	4.81 ± 0.97	19	3.65 ± 0.76	17	3.39 ± 0.95	36.606	1	< 0.001	0.309	0.222	1	0.639	0.003	0.683	1	0.411	0.008	BD < HC, C = M, no significant int
Confident	28	5.88 ± 1.02	22	6.11 ± 0.89	19	5.33 ± 1.17	17	5.11 ± 1.34	10.437	1	0.002	0.113	0.000	1	0.996	0.000	0.906	1	0.344	0.011	BD < HC, C = M, no significant int
Happy	28	5.61 ± 0.97	22	5.97 ± 0.85	19	4.62 ± 0.75	17	4.40 ± 1.15	38.748	1	< 0.001	0.321	0.102	1	0.750	0.001	2.042	1	0.157	0.024	BD < HC, C = M, no significant int
Negative affect	28	1.43 ± 0.54	22	1.26 ± 0.29	19	2.28 ± 0.71	17	2.86 ± 1.05	70.759	1	< 0.001	0.463	1.969	1	0.164	0.023	6.539	1	0.012	0.074	BD > HC, C = M, significant int: BDM > BDC > HCM = HCC
Sad or depressed	28	1.37 ± 0.60	22	1.22 ± 0.44	19	2.26 ± 0.85	17	2.95 ± 1.41	49.675	1	< 0.001	0.377	2.172	1	0.144	0.026	5.052	1	0.027	0.058	BD > HC, C = M, significant int: BDM > BDC > HCM = HCC
Angry or upset	28	1.25 ± 0.50	22	1.17 ± 0.28	19	1.74 ± 0.62	17	2.08 ± 0.95	28.388	1	< 0.001	0.257	0.977	1	0.326	0.012	2.498	1	0.118	0.030	BD > HC, C = M, no significant int
Anxious or nervous	28	1.44 ± 0.57	22	1.26 ± 0.31	19	2.47 ± 0.86	17	2.95 ± 1.24	65.122	1	< 0.001	0.443	0.774	1	0.381	0.009	3.795	1	0.055	0.044	BD > HC, C = M, no significant int
Stressed	28	1.67 ± 0.67	22	1.39 ± 0.36	19	2.66 ± 0.95	17	3.45 ± 1.66	52.796	1	< 0.001	0.392	1.515	1	0.222	0.018	6.449	1	0.013	0.073	BD > HC, C = M, significant int: BDM > BDC > HCM = HCC
Mean 5C-CPT sensitivity^f^	28	0.95 ± 0.04	22	0.97 ± 0.02	19	0.95 ± 0.03	17	0.92 ± 0.08	3.945	1	0.051	0.050	0.284	1	0.596	0.004	3.297	1	0.073	0.042	BD = HC, C = M, no significant int

^a^General linear models for mean affect and mean 5C-CPT sensitivity were without covariates

^b^PSQI is Pittsburgh Sleep Quality Index, a self-rated questionnaire which assesses sleep quality

^c^Global cognitive T-score is calculated from Cognitive Composites Scores, assessing seven cognitive domains

^d^HAM-D is Hamilton Depression Rating Scale which assesses depressive symptoms

^e^YMRS is Young Mania Rating Scale which assesses manic symptoms

^f^5-choice continuous performance test (5C-CPT) is a translational tool to measure cognitive control processes such as attention

^g^C = cycling, M = menopausal, Int = interaction

^h^Race/ethnicity is collapsed into White vs. non-White and compared by diagnostic group (Wald = 0.010; df = 1; *p* = 0.921; Exp(B) = 1.067), reproductive group (Wald = 1.145; df = 1; *p* = 0.285; Exp(B) = 1.855), and their interaction (Wald = 1.857; df = 1; *p* = 0.244; Exp(B) = 0.354) using binary regression

**Fig. 1:**
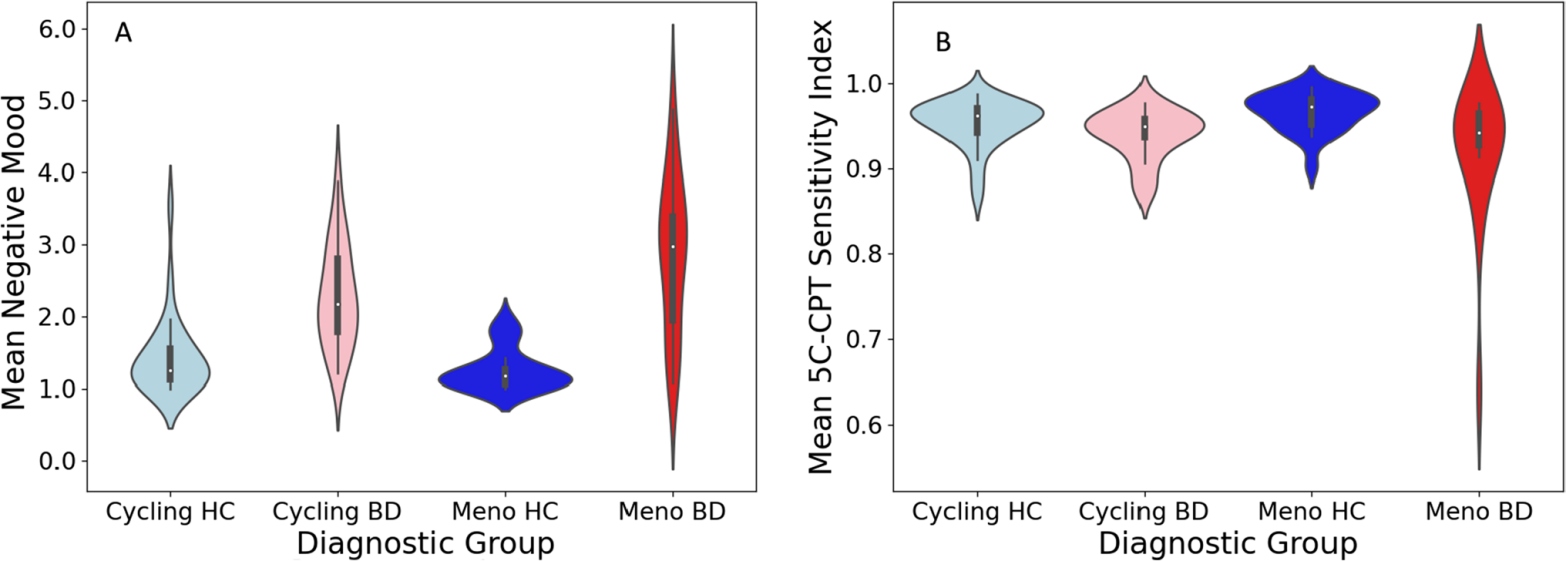
**A** Mean negative mood across cycling HC (cycling healthy comparison), meno HC (menopausal healthy comparison), cycling BD (cycling bipolar disorder), and meno BD (menopausal bipolar disorder). **B** Mean 5C-CPT (5-choice computerized continuous performance test) sensitivity index across four groups

No significant difference was found in 5C-CPT SI, nor was there a main effect of reproductive status (Table 1). Positive affect was lower in BD than HC (F(1,25.2) = 34.7, *p* < 0.001, ${\eta }^{2}$= 0.297), but no diagnosis x menopausal status significant interaction was found. However, negative affect was higher in BD than HC (F(1,31.2) = 70.8, *p* < 0.001, ${\eta }^{2}$= 0.463), and there was a diagnosis x menopausal status interaction (F(1,2.9) = 6.5, *p* = 0.012, ${\eta }^{2}$= 0.074); menopausal women with BD had more negative affect than the other three groups (Fig. 1A), even after considering covariates (F(1,1.9) = 4.0, *p* = 0.050, ${\eta }^{2}$= 0.058). Follow-up analyses found that “sad or depressed” and “stressed” affect in menopausal women with BD was higher compared to other groups (F(1,3.6) = 5.1, *p* = 0.027, ${\eta }^{2}$= 0.058; F(1,5.9) = 6.4, *p* = 0.013, ${\eta }^{2}$ = 0.073); “stressed” in menopausal BD remained significantly higher when considering covariates (F(1,4.5) = 4.3, *p* = 0.043, ${\eta }^{2}$= 0.062). Composite and individual affect differences persisted after sensitivity analysis removing one outlier (negative affect: F(1,2.3) = 5.9, *p* = 0.017, ${\eta }^{2}$= 0.068; sad or depressed: F(1,2.9) = 4.4, *p* = 0.039, ${\eta }^{2}$= 0.052; stressed: F(1,5.1) = 5.8, *p* = 0.018, ${\eta }^{2}$ = 0.067). There was no diagnosis x reproductive status interaction for the 5C-CPT SI (Fig. 1B). Furthermore, there was no significant association between attention performance and negative and positive daily affect scores in either diagnostic (F(1,0.005) = 0.385, *p* = 0.537, ${\eta }^{2}$ = 0.005; F(1,0.025) = 1.995, *p* = 0.162, ${\eta }^{2}$ = 0.026) or reproductive status group (F(1,0.002) = 0.170, *p* = 0.681, ${\eta }^{2}$ = 0.002; F(1,0.004) = 0.290, *p* = 0.592, ${\eta }^{2}$ = 0.004).

## Discussion

This study is the first using EMA to compare daily mood between menopausal and cycling women with and without BD. The results support our hypothesis that menopause is associated with daily negative mood in BD. Contrary to expectations, menopausal women with BD did not experience significantly lower positive mood in their daily life compared to other groups; menopausal women, whether in BD or HC groups, did not exhibit significantly poorer attentional performance.

Our findings are consistent with prior research (Perich et al. 2021; Gilden et al. 2021) and persist after accounting for disparities in age and sleep quality. Collectively, these data support the premise that menopausal status, through mechanisms such as female sex hormone levels that continue to drop and remain low during post-menopause, elevates daily negative affect in BD women. That we did not see higher anxiety in menopausal BD might be due to assessing stress and anxiety separately or because momentary measures may be more representative of daily anxiety and less influenced by memorable high anxiety events than retrospective measures.

Attention was not impaired in BD vs HC in this sample, nor did we observe a relationship to reproductive status or associations that differed by diagnostic group. Our BD participants may have been those with relatively intact cognition. The lack of difference by reproductive status in either HC or BD might be due to the less complex nature of 5C-CPT cognitive test (Hogervorst et al. 2022).

Cognitive performance and daily mood were not significantly related in either group. Our BD patients were generally experiencing only mild mood symptoms at the time of data collection. Prior studies found SI deficits only during BD mania, not when mood symptoms were mild or absent (Young et al 2020). Mild daily negative mood, although elevated somewhat in menopausal BD, might be too subtle to have a noticeable effect on attentional performance.

Study limitations include (1) lack of assessment of reproductive history and hormone levels, (2) inability to compare individuals with different menstrual phases or within menopausal transition, (3) statistical control of age differences across menopausal status, which might not be adequate to isolate the unique effect of menopause, and (4) sample size too small to compare across ethnicities. In general, our findings suggested that women with BD may need additional support for negative mood symptoms during menopause and their treatment should emphasize reduction of depressive symptoms, particularly by alleviating stress.

## Data Availability

Data are available upon request from the author.
